# Comment on Sah et al. Monkeypox and Its Possible Sexual Transmission: Where Are We Now with Its Evidence? *Pathogens* 2022, *11*, 924

**DOI:** 10.3390/pathogens11121417

**Published:** 2022-11-25

**Authors:** Milagros Nilda Vera, Kely Sanca, Segundo Ramos Leon

**Affiliations:** 1 Academic Program of Human Medicine, San Juan Bautista Private University, Chincha Branch, Ica 11000, Peru; 2 Global Health Research Institute, San Juan Bautista Private University, Lima 15067, Peru

After an exhaustive reading of the article “Monkeypox and Its Possible Sexual Transmission: Where Are We Now with Its Evidence?” by Ranjit Sah et al. [1], we infer that monkeypox (MPX) is an incident sexually transmitted infection and considered today a public health emergency. MPX is a zoonosis with transmission from person to person. The pathogenesis and exact transmission mechanisms of MPX are still under investigation, but it has been reported that most infections occur in men who have sex with men (MSM) [2].

In Peru, as of 16 October 2022, around 2913 cases have been confirmed nationwide, with the department of Lima being the one that reported the highest number of cases. In this scenario, the National Institute of Health (INS) has confirmed community transmission of MPX in Peru. It has been noted that the reported cases are associated with casual sexual contact in clubs and saunas, where sexual intercourse with multiple partners is observed, especially among bisexual and homosexual men [3]. 

Another point to consider is the concomitant infection of monkeypox with the human immunodeficiency virus (HIV). The Peruvian Minister of Health (MINSA) estimates that until 3 October, 5036 new cases of HIV were reported for 2022, with males being the one with the highest rate (80.2%). According to the previous epidemiological update, as of August 20, amongst all patients diagnosed with monkeypox, 62.8% (671 out of 1068) have HIV and 21.7% (232) did not take their ART medications, making Peru the country with the largest number of patients who have this co-infection. 

Regarding the transmission of MPX, the article reports 54 cases of monkeypox presented in a health center in Great Britain, where it was observed that 100% of the cases were homosexual patients. This raises the fact about the sexual transmission of this disease. 

Therefore, monkeypox is transmitted by sexual contact, making this an important route for spreading the infection. This is being supported by laboratory studies from various scientific centers around the world and, it should be noted that Peru has the same behavior, where researchers from the INS and MINSA reported that 95% or even more of the cases occur in men who have sex with men (MSM) and bisexuals [4].
